# The experience of the COMRE group (REctal COMmittee): can magnetic resonance imaging and endosonography really help the clinical pathway after NCRT in rectal cancer?

**DOI:** 10.1097/JS9.0000000000000579

**Published:** 2023-07-05

**Authors:** Stefano Scabini, Chiara Romana, Marina Sartini, Ali Attieh, Ciro Marrone, Maria L. Cristina, Maria C. Parodi

**Affiliations:** a Oncological Surgical Unit; b Interventional Gastroenterology Unit, IRCCS Ospedale Policlinico San Martino; c Department of Health Sciences, University of Genova; d Hospital Hygiene Unit, Galliera Hospital; e Oncological and Interventional Radiology Unit, IRCCS Ospedale Policlinico San Martino, Genoa, Italy

**Keywords:** agreement, diagnostic accuracy, magnetic resonance imaging, rectal endosonography

## Abstract

**Background:**

MRI and rectal endosonography (EUS) are routinely used for preoperative tumor staging and assessment of response to therapy in patients with rectal cancer. This study aimed to evaluate the accuracy of the two techniques in predicting the pathological response compared to the resected specimen and the agreement between MRI and EUS and to define the factors that could affect the ability of EUS and MRI to predict pathological responses.

**Materials and methods:**

This study included 151 adult patients with middle or low rectal adenocarcinoma treated with neoadjuvant chemoradiotherapy, followed by curative intent elective surgery in the Oncologic Surgical Unit of a hospital in the north of Italy between January 2010 and November 2020. All patients underwent MRI and rectal EUS.

**Results:**

The accuracy of EUS to evaluate the T stage was 67.48%, and for the N stage was 75.61%; the accuracy of MRI to evaluate the T stage was 75.97%, and that for the N stage was 51.94%. The agreement in detecting the T stage between EUS and MRI was 65.14% with a Cohen’s kappa of 0.4070 and that for the evaluation of the lymph nodes between EUS and MRI was 47.71% with a Cohen’s kappa of 0.2680. Risk factors that affect the ability of each method to predict pathological response were also investigated using logistic regression.

**Conclusions:**

EUS and MRI are accurate tools for rectal cancer staging. However, after Radiotherapy - Chemotherapy RT-CT, neither method is reliable for establishing the T stage. EUS seems significantly better than MRI for assessing the N stage. Both methods can be used as complementary tools in the preoperative assessment and management of rectal cancer, but their role in the assessment of residual rectal tumors cannot predict the complete clinical response.

## Introduction

Highlights

- We evaluated the accuracy of MRI and rectal endosonography (EUS) in predicting rectal cancer response to therapy compared to the resected specimen.
- We also assessed the agreement between MRI and EUS and attempted to define the factors that could affect the ability of EUS and MRI to predict pathological responses.
- We found that EUS and MRI are accurate tools for rectal cancer staging; however, after Radiotherapy - Chemotherapy, neither method is reliable for establishing the T stage and EUS seems significantly better than MRI for assessing the N stage.
- Both methods can be used as complementary tools in the preoperative assessment and management of rectal cancer, but their role in the assessment of residual rectal tumors cannot predict the complete clinical response.

Rectal cancer is the third most common cancer and the second-leading cause of cancer death globally^1^. Although there is an overall trend of decreased incidence of colorectal cancer and mortality, the incidence of rectal cancer in individuals younger than 50 years has been exponentially increasing, with rates estimated to rise to 124.2% in individuals aged 20–34 years by 2030 in the USA^2^. The treatment for rectal cancer is evolving, and determining the ‘ideal’ treatment is a multifaceted process that requires consideration of patient preferences, patient performance status, tumor characteristics, the intent of the surgery, and outcomes.

The encouraging oncological results of the watch and wait approach^3,4^ increase the need for accurate clinical response evaluation to identify patients with a complete or near-complete clinical response to chemoradiotherapy (CRT)^5^. Moreover, as rectal cancer treatment becomes more precise, high-resolution MRI and rectal ultrasonography have been established to identify important tumor characteristics that help guide management^6^.

The ability to identify ‘prognostic factors’ such as tumor submucosal invasion, extramural venous invasion, threatened margins, and node involvement, has introduced the concept of decision-making based on preoperative staging information. According to TMN staging, neoadjuvant CRT (nCRT) and adjuvant chemotherapy are recommended for locally advanced disease (LARC) T3–4, node-negative (N0), or node-positive disease without distant metastasis (N1–2, M0). For patients with low-risk LARC undergo surgery after a preoperative short course of radiation therapy; surgery alone is recommended for early rectal cancer. Endosonography (EUS) and MRI are routinely used for preoperative tumor staging and to assess response to therapy. Assessment of wall invasion and metastatic lymph nodes is better than other imaging modalities and is so efficacious that treatment might be altered according to endosonographic staging^7^. However, only a few studies have analyzed the accuracy and agreement of EUS and MRI in predicting the response to neoadjuvant therapy.

This study aimed to evaluate the accuracy of the two restaging techniques (EUS and MRI) after nCRT in the prediction of the pathologic response, evaluate the agreement between the two techniques, and compare their results to the pathological response as determined using a resected specimen. The secondary aim was to define factors (patient- or tumor-related) that can affect the ability of EUS and MRI to predict pathological responses.

## Materials and methods

### Patients

This retrospective study enrolled 151 patients (age, >18 years) from 327 consecutive patients with stage I, II, or III middle or low rectal adenocarcinoma who underwent total mesorectal excision at the tertiary referral center (Oncologic Surgical Unit, which performs more than 150 colorectal resections per year) of a hospital in the north of Italy. All enrolled patients had histologically confirmed locally advanced rectal adenocarcinoma (clinical T3-T4 N0M0 or T [any] N + M0) and were treated with nCRT followed by curative intent elective surgery between January 2010 and November 2020. The patients were examined using MRI and rectal EUS according to the most recent guidelines^8,9^. The two techniques were performed at time zero (diagnosis) and 2 months after CRT.

All MRI reports were presented according to international standards, validated by a specialized lower gastrointestinal radiologist, and performed by a single expert endoscopist. The depth of tumor invasion into the rectal wall (T stage) and lymph node involvement (N stage) were determined according to the ultrasonographic classification proposed by Hildebrandt and Feifel^10^. The initial stage was determined using EUS and MRI according to the TMN classification of malignant tumors. The initial radiological stage was higher than T2N0 for all patients.

The study was conducted according to the guidelines of the Declaration of Helsinki, and approved by the Institutional Review Board (Ethics Committee) (protocol code 364REG2015 - 02/09/2015).

### Statistical analysis

Excel (Microsoft Office 365, Microsoft Corporation) was employed to keep track of patient-related records. A descriptive analysis of the data was conducted. The agreement between MRI and EUS was assessed using the *χ* ^2^-test and Cohen’s Kappa test. A Kappa less than 0.01 indicates zero agreement; 0.01–0.20, poor; 0.21–0.40, modest; 0.41–0.60, moderate; 61–0.80, good; and 0.81–1.00, excellent.

We evaluated the accuracy of EUS and MRI to assess the pathological response after CRT, considering T stage and N stage for each technique using MedCalc diagnostic test calculator (MedCalc Software Ltd.).

Stepwise logistic regression models, were used to estimate the odds ratio (OR) and 95% CI to identify the factors (sex, age, BMI, N stage, T stage, or tumor location) that affect EUS and MRI in the correct way (True Positive/True Negative).

The significant level was set to *P*<0.05 for all tests.

All analyses were performed using STATA® SE14 (StataCorp LP).

## Results

The mean age of the patients included in the study was 64.63±11.54 years (range, 28–84 years). Patients presented with histologically confirmed middle and distal rectal tumors were 48.34 and 51.66%, respectively. Of the patients, 61.59% were men (Table 1).

**Table 1:** Baseline characteristics of 151 patients.

Age (years old); median (IQR)	66 (57–74)
Sex (male), *N* (%)	93 (61.59)
BMI, median (IQR)	24.22 (23.03–25.71)
Imaging T stage *N* (%)	
** **T0	35 (23.18)
** **T1	11 (7.28)
** **T2	36 (23.84)
** **T3	64 (42.38)
** **T4	5 (3.31)
Imaging N stage	
** **N0	117 (78.00)
** **N1	26 (17.33)
** **N2	5 (3.33)
** **Nx	2 (1.33)
Rectal Location, *N* (%)	
** **Distal	78 (51.66)
** **Middle	73 (48.34)

At restaging after nCRT, 15.23% of patients were classified as T0, and only 7.95% as Tx.

As for tumor staging, 13.91% (21) were stage 0, 25.17% (38) were stage I, 31.13% (47) were stage II, 20.53% (31) were stage III, and 9.27% (14) were stage X.

One hundred patients (66.23%) underwent anterior resection; in 39 (25.82%) patients, abdominoperineal excision was also performed; six (3.97%) patients underwent transanal TME (TaTME), three patients (1.99%) underwent Hartmaan’s surgery, four patients underwent colorectal anastomosis (2.65%) and coloanal (0.66%) anastomosis. The average postsurgery hospitalization was 10.23±6.88 (2–57 days; median, 8; IQR, 7–12).

Surgery was performed ~94.39±43.37 days (range, 10–450 days) after completion of the chemoradiation protocol, and restaging with EUS and MRI was performed prior to surgery (usually 1–2 weeks).

The MRI and ultrasonography tumor regression grades were revised and compared with the specimen examination for all patients.

### Accuracy

We evaluated the accuracy of EUS and MRI in assessing the pathological response after CRT, considering the T and N stages for each technique. The Accuracy of EUS to evaluate the T stage was 67.48% and that for the N stage was 75.61%; the accuracy of MRI to evaluate the T stage was 75.97% and that for the N stage was 51.94% (Table 2).

**Table 2:** (a) Diagnostic accuracy of EUS T and RMN T. (b) Diagnostic accuracy of EUS N and RMN N.

	Accuracy of EUS T	Accuracy of RMN T
*N° of patients*	123	129
*True positive*	67	88
*False positive*	11	21
*False negative*	29	10
*True negative*	16	10
*Sensitivity*	69.79% (59.57–78.75%)	89.80% (82.03–95.00%)
*Specificity*	59.26% (38.80–77.61%)	32.26% (16.68–51.37%)
*Positive Predictive Value*	85.90% (79.14–90.72%)	80.73% (76.51–84.35%)
*Negative Predictive Value*	35.56% (26.29–46.05%)	50.00% (31.48–68.52%)
*Diagnostic Accuracy*	67.48% (58.45–75.65%)	75.97% (67.66–83.05%)
	Accuracy of EUS N	Accuracy of RMN N
*N° of patients*	123	129
*True positive*	7	24
*False positive*	12	57
*False negative*	18	5
*True negative*	86	43
*Sensitivity*	28.00% (12.07–49.39%)	82.76% (64.23–94.15%)
*Specificity*	87.76% (79.55–93.51%)	43.00% (33.14–53.29%)
*Positive Predictive Value*	36.84% (20.40–57.03%)	29.63% (24.92–34.82%)
*Negative Predictive Value*	82.69% (78.73–86.05%)	89.58% (78.97–95.17%)
*Diagnostic Accuracy*	75.61% (67.05–82.90%)	51.94% (42.97–60.81%)

The agreement in accuracy between EUS and MRI for the T stage was also evaluated (Table 3).

**Table 3:** Agreement between EUS T and RMN T.

	RMN T			
EUS T	True positive (%)	False positive (%)	False negative (%)	True negative (%)
True positive	52 (86.67)	1 (1.67)	5 (8.33)	2 (3.33)
False positive	0 (0.00)	9 (100.00)	0 (0.00)	0 (0.00)
False negative	21 (84.00)	0 (0.00)	4 (16.00)	0 (0.00)
True negative	0 (0.00)	9 (60.00)	0 (0.00)	6 (40.00)

The agreement in detecting the T stage between EUS and MRI was 65.14% (expected agreement 41.21%), with a Cohen’s Kappa of 0.4070 (95% CI, 0.2705–0.5435) (*P*<0.001) (Table 4).

**Table 4:** Agreement e Kappa di Cohen between EUS T e RMN T.

	Agreement (%)	Kappa	SE	*P*	IC 95%
All	65.1	0.4070	0.0565	0.0000	0.2705–0.5435

The agreement in accuracy between EUS and MRI for the N stage is shown in Table 5.

**Table 5:** Agreement between EUS N and RMN N.

	RMN N			
EUS N	True positive (%)	False positive (%)	False negative (%)	True negative (%)
*True positive*	6 (85.71)	1 (14.29)	0 (0.00)	0 (0.00)
*False positive*	0 (0.00)	8 (88.89)	0 (0.00)	1 (11.11)
*False negative*	14 (82.35)	0 (0.00)	3 (17.65)	0 (0.00)
*True negative*	0 (0.00)	39 (51.32)	2 (2.63)	35 (46.05)

The agreement between the two methods for the evaluation of the lymph nodes was 47.71% (expected agreement, 28.56%), with a Cohen’s Kappa of 0.2680 (*P*<0.001) (Table 6).

**Table 6:** Agreement e Kappa di Cohen between EUS N and RMN N.

	Agreement (%)	Kappa	SE	*P*-value	IC 95%
All	47.7	0.2680	0.0439	0.0000	0.1684–0.3676

### Logistic regression

Upon investigating the risk factors related to the patient or the tumor, which have an impact on the ability of each method to predict pathological response, no risk factor influences the predictive ability of EUS in predicting the T stage. Whereas in predicting the N stage (N0 vs. N+ [0 vs. 1]), more false positive/false negative outcomes (OR 19.0; IC 95% 6.21–58.11; *P*=0.000) were noted.

For MRI-T, the T stage (T0 vs. T+) was better able to predict a TP/TN outcome (OR 0.063; IC 95% 0.024–0.169; *P*=0.000). For MRI-N, the factors that influenced the test outcome were N stage (N0 vs. N+), T stage (T0 vs. T+), and sex (male 0 vs. female 1) (Table 7).

**Table 7:** Factor influencing the ability of each method predict pathological response.

	Odds ratio	SE	z	*P*	IC 95%
EUS N					
* N stage*	19.000	19.838	5.16	0.000	6.21–58.11
RMN T					
*T stage*	0.063	0.032	−5.50	0.000	0.024–0.169
RMN N					
*N stage*	0.088	0.053	−4.01	0.000	0.027–0.289
*T stage*	2.968	1.362	2.37	0.018	1.207–7.298
*Sex*	0.438	0.181	−2.00	0.045	0.195–0.982

## Discussion

Radiological assessment with MRI and ultrasonographic assessment with rectal EUS before surgery for detecting pathological rectal cancer after nCRT were reported in our study. The agreement between MRI and EUS to detect tumor regression was analyzed.

The results suggest moderate agreement between radiologic and ultrasonography techniques and the pathologic assessment when the ‘T stage’, known as the stage of layers invasion, is evaluated, with a Cohen’s Kappa of 0.4070 (95% CI 0.2705–0.5435) (*P*<0.001). For the evaluation of lymph node response, on comparing the accuracy of rectal ultrasonography and MRI, we consider that there is a suboptimal agreement between the two methods with a Cohen’s kappa of 0.2680 (*P*<0.001).

Our study agrees with a recent meta-analysis published by Memon *et al.* ^11^, which showed that MRI treatment response has poor accuracy for T stage prediction (52 vs. 75.97% in our study) and EUS T stage restaging accuracy was non-significantly higher (65 vs. 67.48% in our study). In this meta-analysis, the restaging with MRI and EUS was similar in terms of prediction of nodal status: the accuracy of both investigations was 72%, with over-staging and under-staging occurring in 10–15%, as shown in Table 2. In our study, we report an accuracy of 75.61% for EUS N and 51.94% for MRI-N.

In a large cohort by Maretto *et al.* ^12^, complete responders could be detected only partially on restaging with MRI and EUS; the accuracy in predicting T status on MRI was 77% and that for EUS was 64%, similar to that in our series. In predicting N status (N-negative vs. N-positive), they found an accuracy of 61% for EUS and 65% for MRI.

The presence of edema, inflammation, necrosis, and fibrosis that cannot be distinguished from the residual tumor cause difficulty in the post-CRT assessment of rectal cancer for a radiologist or gastroenterologist. Some radiologists consider areas of very low signal intensity as fibrosis whereas others consider these to be areas of residual tumor^13^. Conversely, EUS alone is not sufficient to obtain accurate preoperative staging, particularly for bulky tumors, tumors with stenosis, and tumors located in the upper third of the rectum; it is necessary to have another evaluation method, such as the MRI. MRI can evaluate the mesorectal fascia, which plays an important role in preoperative evaluation because it can show the distance of the tumor from the mesorectal fascia and help determine the type of surgery.

The secondary aim of our study was to identify factors (patient-related or tumor-related) affecting the ability of EUS and MRI to predict pathological regression. Notably, we found that for EUS, no risk factor influences the predictive ability of the test, whereas, for EUS N, the N stage [N0 vs. N+ (0 vs. 1)] was able to predict a False Positive/False Negative outcome.

For MRI-T, the T stage (T0 vs. T+) was better able to predict true positive/true negative outcomes. For MRI-N, the factors that could affect the test outcome were the N stage (N0 vs. N+), the T stage (T0 vs. T+), and sex (male 0 vs. female 1).

These data can be explained by the fact that the more the tumor invades the perirectal fat beyond the muscularis propria, the more likely it is that MRI and EUS assessments will be concordant with the pathological examination. Furthermore, the deeper the extension of the tumor in the mesorectal fat, the more likely it is to have positive lymph nodes and the easier it is to appreciate the regression of tumors after nCRT.

This study compared rectal EUS and MRI for staging rectal cancer, after neoadjuvant radiotherapy-chemotherapy: both EUS and MRI offered poor diagnostic performance in the assessment of T and N stages compared to the ‘gold standard’, histological examination of surgical specimens^14^. In the present study, compared to MRI, EUS offered significantly superior diagnostic accuracy in assessing nodal involvement, whereas MRI was more accurate for patients with more advanced disease (on the ‘T’ evaluation), with a higher sensitivity (89.8 vs. 69.7%).

### Limitation

This is a retrospective study, with no randomization. It also includes a limited number of patients who were nevertheless studied in a monocentric and homogeneous manner; this last point is very important because it allows us to limit the operator-dependent bias.

A serious limitation of the study is the sample size that was small to draw definitive conclusions and although the results were limited to a single center.

Finally, this is a pilot study to see if the two methods (MRI and EUS) can help the doctor determine the subsequent diagnostic and therapeutic approach.

## Conclusion

EUS and MRI are accurate for rectal cancer staging. However, after Radiotherapy - Chemotherapy, neither method is reliable for establishing the T stage. EUS seems to be significantly better than MRI for assessing the N stage. The present study revealed moderate agreement between MRI and EUS in the assessment of the T stage but only suboptimal agreement for the evaluation of lymph node response. Therefore, both methods can be used as complementary tools in the preoperative assessment and management of rectal cancer; however, their role in the assessment of residual rectal tumors cannot predict the complete clinical response. Research needs to focus not only on this, but also on other prognostic response factors, such as molecular biological disease factors^15^.

## Ethical approval

Institutional Review Board (or Ethics Committee) of IRCCS San Martino IST of Genoa (Italy) (protocol code 364REG2015 - 02/09/2015).

## Sources of funding

None.

## Author contribution

S.S. and C.R.: conceptualization; S.S. and M.S.: methodology; S.S. and M.S.: software; M.S.: validation; M.S. and M.L.C.: formal analysis; A.A. and C.M.: investigation; S.S., M.L.C., and M.C.P.: resources; C.R. and M.S.: data curation; S.S., C.R., and M.S.: writing—original draft preparation; S.S., C.R., and M.S.: writing—review and editing. All authors have read and agreed to the published version of the manuscript.

## Conflicts of interest disclosure

The authors declare that they have no financial conflict of interest with regard to the content of this report.

## Guarantor

Stefano Scabini, Chiara Romana, Maria Luisa Cristina and Marina Sartini.
